# Computationally-efficient statistical design and yield optimization of resonator-based notch filters using feature-based surrogates

**DOI:** 10.1038/s41598-023-42056-7

**Published:** 2023-09-08

**Authors:** Slawomir Koziel, Tanveerul Haq

**Affiliations:** 1 Engineering Optimization and Modeling Center, Reykjavik University, 101 Reykjavik, Iceland; 2 Faculty of Electronics, Telecommunications and Informatics, Gdansk University of Technology, 80-233 Gdansk, Poland

**Keywords:** Physics, Electronics, photonics and device physics

## Abstract

Modern microwave devices are designed to fulfill stringent requirements pertaining to electrical performance, which requires, among others, a meticulous tuning of their geometry parameters. When moving up in frequency, physical dimensions of passive microwave circuits become smaller, making the system performance increasingly susceptible to manufacturing tolerances. In particular, inherent inaccuracy of fabrication processes affect the fundamental operating parameters, such as center frequency or bandwidth, which is especially troublesome for narrow-band structures, including notch filters. The ability to quantify the effects of tolerances, and—even more—to account for these in the design process, are instrumental in making the designs more reliable, and to increase the likelihood that adequate operation is ensured despite manufacturing errors. This paper proposes a simple yet computationally efficient and reliable procedure for statistical analysis and yield optimization of resonator-based notch filters. Our methodology involves feature-based surrogate models that can be established using a handful of training data points, and employed for rapid evaluation of the circuit fabrication yield. Furthermore, a yield optimization procedure is developed, which iteratively sets up a sequence of feature-based models, constructed within local domains relocated along the optimization path, and uses them as predictors to find a robust (maximum yield) design at a low computational cost. The presented approach has been demonstrated using two complementary split ring resonator (CSRR)-based notch filters. The cost of statistical design is about a hundred of EM simulations of the respective filter, with yield evaluation reliability corroborated through EM-based Monte Carlo analysis.

## Introduction

Microwave notch filters are essential components of industrial microwave and millimeter-wave systems. Microwave filters are continually gaining momentum as they play a crucial role in frequency-agile RF interfaces and payloads, driven by rapid evolution in the wireless electronics industry^1^ and the growing need for more efficient and adaptive multichannel communication systems^2^. The RF filter market is predicted to increase at a rate of more than ten percent per year, surpassing $168 million by 2027^3^, owing mostly to important industrial sectors such as radar systems, and telecommunication. Microwave notch filters are used in communication systems to prevent interference from unwanted signals. Furthermore, these filters are used in the rapid prototyping industry to measure angular displacement^4^, proximity and rotation^5^.

Design of microwave filters can be challenging due to several factors, including frequency dependence, bandwidth, insertion loss, temperature stability, manufacturing tolerances, size, and weight. Depending on the specific application, microwave notch filters have been designed utilizing a variety of techniques. Notch filters with cavity resonators^6^, waveguide structures^7^, and microstrip transmission lines^8^ are just a few of the popular techniques. Waveguide and cavity-based notch filters exhibit certain advantages such as a high quality factor, minimum insertion loss, high power handling, and steady performance, but also disadvantages such as a limited tuning range, a bulky size, higher manufacturing costs, as well as integration difficulties^9^. On the other hand, microstrip notch filters feature small size, low cost, broad frequency ranges of operation, and overall good performance. Their disadvantages include a limited power handling capability, temperature sensitivity, limited bandwidth, susceptibility to external interference, and tuning difficulty^10^. Recently, complementary metamaterial resonators have been found to offer a promising approach to design microwave notch filters. Their unique electromagnetic properties make them well-suited for applications in microwave communication systems and other areas where precise filtering of microwave radiation is required^11^. However, the performance of complementary metamaterial-based microwave notch filters depends on the precision of their fabrication and calibration^12^. Microwave notch filters based on complementary metamaterial resonators can be rendered unusable by even minute changes in geometric dimensions. The microwave filters presented in^13–16^, have been fabricated using various manufacturing techniques and measured using different vector network analyzers. However, a discrepancy of up to four percent has been observed between the simulation-based predictions and the experimental validation. To minimize these differences, pre- or post-fabrication tuning is required, especially for high-frequency resonators. At the same time, understanding the effects of manufacturing inaccuracies is a prerequisite for realizing high-performance designs. Preferably, quantification of geometrical dimension discrepancies and other types of uncertainties (e.g., pertinent to material parameters) should be incorporated into the design process so as to render circuits that are more robust and immunized against tolerances.

Manufacturing tolerances are stochastic in nature, and can therefore be described using appropriate probability distributions. Consequently, their evaluation requires statistical analysis^17^, which is a computationally expensive endeavor if carried out using electromagnetic (EM) simulation models. In particular, direct EM-based Monte Carlo analysis^18^ typically requires many hundreds of system evaluations, which often turns prohibitive. Accelerated methods rely on simplifications (e.g., worst case analysis^19^), fast replacement models (surrogates), e.g., neural networks^20^, polynomial chaos expansion^21,22^, dimensionality reduction (e.g., principal component analysis^23^, variable-fidelity simulations^24^ or physics-based modeling^25^).

Accurate quantification of the effects of uncertainties is a prerequisite for robust design (design centering^26^, tolerance-aware design^27^), which attempts to improve the system immunity to fabrication or other types of tolerances. The underlying task may be formulated in various ways (e.g., reduction of the system response sensitivity to parameter deviations, maximization of yield^28^, maximization of tolerance hypervolume^29^), although in the case of microwave components, the most common approach is the improvement of the fabrication yield. The latter is a likelihood that given performance specifications will be satisfied under the assumed parameter deviations. Similarly, as for statistical analysis, practical robust design involves surrogate modeling techniques^30–34^. The bottleneck is a large number of EM analyses necessary to set up a reliable model, which has to be valid over a relatively large portion of the parameter space due to the expected design relocation in the course of the optimization process. A possible way of alleviating this issue is sequential approximate optimization^35^, where the surrogate model is iteratively constructed in a local domain that moves along the optimization path. Another possibility is a response feature technology^36^. Therein, design problem is reformulated in terms of so-called characteristic points (response features) of the system outputs, which are normally in weakly-nonlinear relationships with the geometry parameters. Inherent regularization offered by this approach facilitates a construction of accurate behavioral models at low computational expenses^37^.

In this paper, we address the problem of rapid uncertainty quantification and yield optimization of resonator-based notch filters using feature-based surrogate models. A set of characteristic points accounting for the operating parameters of the notch filter is introduced, which are extracted from EM-simulated transmission responses, and demonstrated to be in weakly-nonlinear relationship with the circuit geometry parameters. Subsequently, a reliable behavioral model is constructed at the level of response features using a handful of training data samples, and shown to exhibit a remarkable predictive power, both in terms of generalization and extrapolation, as compared to conventional data-driven modeling methods. The feature-based model is applied to evaluate the filter fabrication yield, and to carry out yield optimization upon being embedded in the sequential approximation optimization loop. Two examples of resonator-based notch filters are considered as demonstration case studies, a circular and square complementary split ring resonator (SCRR)-based ones. Maximum yield designs are obtained at the computational costs of merely about a hundred of EM analyses of the respective structures. The observed yield improvements are considerable (from about 80 percent to well over 90 percent, depending on the design scenario). At the same time, the accuracy of yield estimation is excellent as corroborated using direct EM-driven Monte Carlo analysis. The proposed approach can be used to achieve pre-fabrication robustness improvement of microstrip resonator-based notch filters at extremely low computational expenses.

## Resonator-based notch filters: case studies

This section recalls the background information about microstrip resonator-based notch filters, and discusses two specific structures to be considered case studies in the later parts of this paper.

### Resonator-based notch filters

**Figure 1:**
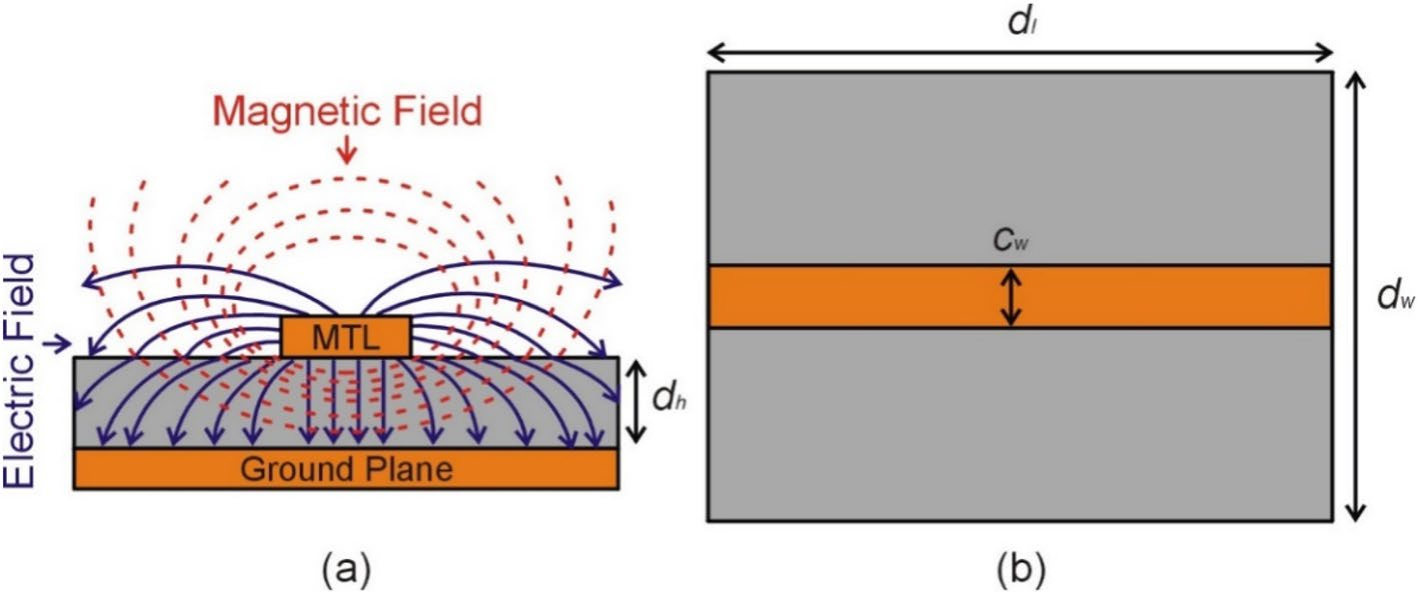
(**a**) The direction of electric and magnetic fields generated by the microstrip transmission line (MTL) coupled to a ground plane, (**b**) dimensions of MTL and a dielectric substrate.

Microwave notch filters based on complementary resonators are a type of structures that utilize the resonant behavior of complementary metamaterial to suppress or attenuate a specific frequency or range of frequencies. In order to design planar microwave notch filters, complementary resonators are etched in the ground plane below the microstrip transmission line (MTL), which is the region with the highest electric field strength, and in an axial direction, as illustrated in Fig. 1a. In this work, a 50-Ω MTL is designed on a dielectric substrate (RO4003C) with relative permittivity *ε*_r_ = 3.38 ± 0.05, *d*_*l*_ = 30 mm, *d*_*w*_ = 25 mm, and *d*_*h*_ = 0.813 mm, as shown in Fig. 1b. The impedance of the MTL can be calculated using the following equations^38^:

1 $$\varepsilon_{re} = \frac{{\varepsilon_{r} + 1}}{2} + \frac{{\varepsilon_{r} - 1}}{2}\left( {1 + 12\frac{{s_{h} }}{{m_{w} }}} \right)^{ - 0.5}$$

2 $$Z_{c} = \frac{\eta }{{\sqrt {\varepsilon_{re} } }}\left\{ {\frac{{m_{w} }}{{s_{h} }} + 1.393 + 0.677\ln \left( {\frac{{m_{w} }}{{s_{h} }} + 1.4444} \right)} \right\}^{ - 1}$$

where the effective dielectric constant of the transmission line is *ε*_*re*_ = 2.67 and the impedance of wave in free space is *η* = 120π Ω, and the width of the microstrip is *c*_*w*_ = 1.88 mm.

The basic structure of complementary resonators consists of two resonators, each of which is designed to have an electric dipole moment that cancels out the electric dipole moment of the other resonator. This cancellation leads to a null or notch in the transmission response of the filter at the resonant frequency. The bandwidth and depth of the notch can be adjusted by modifying the resonators’ geometrical properties, such as their size, shape, and spacing.

### Case studies: circular and square CSRR filters

Consider the following two basic complementary metamaterial resonators, a circular complementary split ring resonator (CC-SRR)^39^, and a square complementary split ring resonator (SC-SRR)^40^.

**Figure 2:**
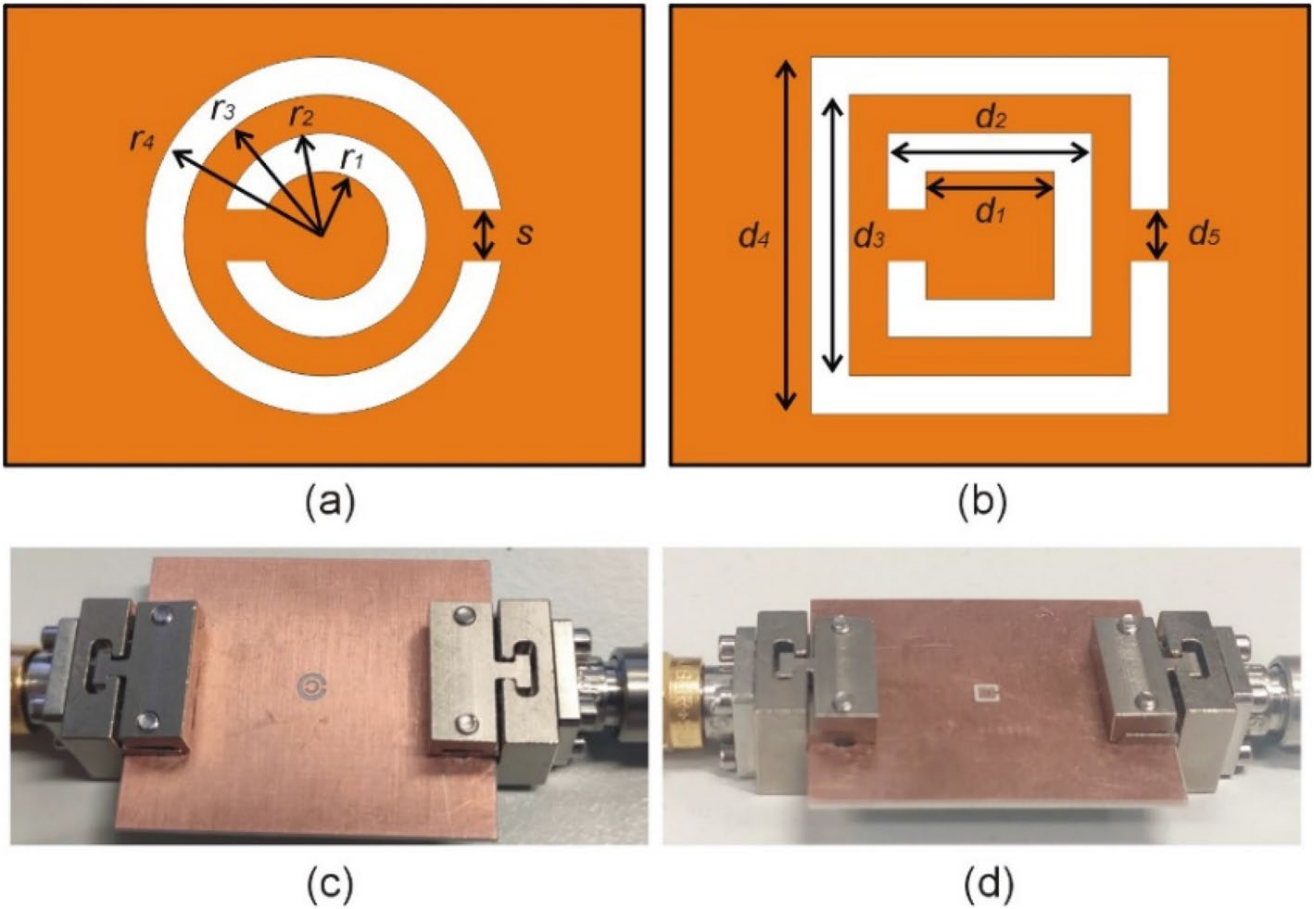
Resonator-based notch filters: (**a**) geometry of the circular complementary split ring resonator (CC-SRR), (**b**) geometry of the square complementary split ring resonator (SC-SRR), (**c**) fabricated microwave notch filter based on CC-SRR (**d**) fabricated microwave notch filter based on SC-SRR.

Both are used to design two microwave notch filters, which will serve as case studies in "Surrogate-assisted statistical design of notch filters using response features" and "Demonstration examples" sections of this work. Five variables are used to describe the geometry of CC-SRR (*r*_1_, *r*_2_, *r*_3_, *r*_4_, and *s*) and SC-SRR (*d*_1_, *d*_2_, *d*_3_, *d*_4_, and *d*_5_) as shown in Fig. 2a and b, respectively. For the sake of illustration, the geometric parameters of both resonators are optimized to achieve a 15 GHz resonance frequency with maximum notch depth. CC-SRR optimization results in *r*_1_ = 0.41 mm, *r*_2_ = 0.59 mm, *r*_3_ = 0.87 mm, *r*_4_ = 1.23 mm, and *s* = 0.18 mm. The resonant frequency is 15 GHz, and the notch depth of − 26.65 dB is obtained by optimization of CC-SR. The optimization of SC-SRR yields the following dimensions *d*_1_ = 0.82 mm, *d*_2_ = 1.05 mm, *d*_3_ = 1.42 mm, *d*_4_ = 2.07 mm, and *d*_5_ = 0.34 mm. The simulated resonance frequency is 15 GHz, and the observed notch depth is − 26.05 dB.

### Filter prototyping: manufacturing tolerances

The optimized microwave notch filters have been manufactured by chemical etching on a double-sided copper-clad laminated (17.5 μm) RO4003C substrate, as illustrated in Fig. 2c and d, respectively.

**Figure 3:**
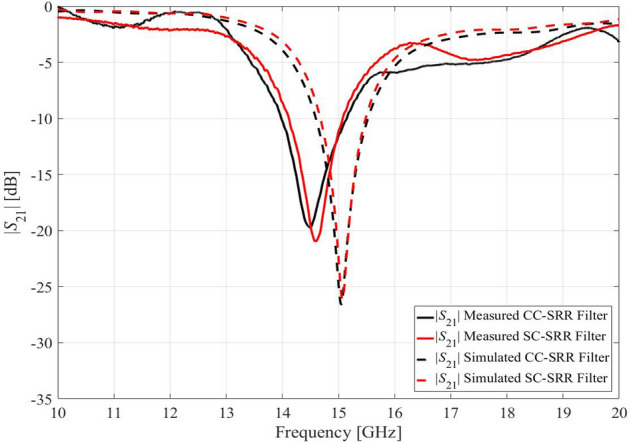
Transmission coefficients |*S*_21_| of the simulated and measured microwave notch filters based on CC-SRR and SC-SRR.

The simulated and measured results for both microwave notch filters are shown in Fig. 3. The measured resonant frequency of the CC-SRR microwave notch filter is 14.51 GHz with a notch depth of − 19.83 dB, indicating a difference of 0.49 GHz between simulation and measurement. The SC-SRR microwave notch filter's measured resonant frequency is 14.62 GHz with a notch depth of − 21.2 dB, showing a 0.38 GHz difference between simulation and measurement. These discrepancies illustrate the effects of manufacturing tolerances elaborated on in "Introduction" section, although the frequency shifts are—in part—due to inaccurate simulation process. The filters of Fig. 2 will be used as demonstration case studies to explain and illustrate the robust design procedure discussed in the remaining part of this article.

## Surrogate-assisted statistical design of notch filters using response features

This section introduces the proposed statistical design approach. We start by discussing design specifications for the notch filters considered in this work as well as design optimization procedure ("Performance specifications: nominal design and design optimization" section), followed by a definition of the fabrication yield in the context of notch filters ("Fabrication yield: definition and numerical estimation" section), as well as formulating the robust design task ("Robust design and its challenges" section). The algorithmic tools developed to carry out yield maximization in a computationally-efficient manner, i.e., feature-based surrogate models and surrogate-assisted framework involving sequential approximate optimization, are introduced in "Feature-based surrogate modeling of notch filters" and "Surrogate-assisted yield estimation and optimization by feature-based models" section, respectively.

### Performance specifications: nominal design and design optimization

The concept of design optimality depends on the performance specifications imposed upon the circuit at hand. For the purpose of subsequent considerations, let us assume that *f*_*t*_* − B*/2 ≤ *f* ≤ *f*_*t*_ + *B*/2 be the range of frequencies *f* of interest, over which the filter transmission coefficient |*S*_21_(***x***,*f*)| should not exceed *L*_max_ (e.g., − 15 dB), where *B* is the target operating bandwidth; *f*_*t*_ is the target center frequency. We also assume that this condition is fulfilled at the nominal design ***x***^(0)^, i.e., we have

3 $$\left| {S_{{{21}}} \left( {x^{(0)} ,f} \right)} \right| \le L_{{{\text{max}}}} {\text{for}}\;{\text{all}}\;f \in F = \left[ {f_{t} {-}B/{2},f_{t} + B/{2}} \right]$$

The nominal design itself, is assumed to be obtained by allocating the filter resonance at the prescribed (target) frequency *f*_*t*_ and, simultaneously, by minimizing the level of transmission |*S*_21_| at *f*_*t*_. We use the following notation:

- ***x*** = [*x*_1_ … *x*_*n*_]^*T*^—a vector of designable (geometry) parameters of the filter;
- *f*_0_(***x***)—resonant frequency at design ***x***;
- *L*_0_(***x***) − |*S*_21_| level at *f*_0_ and design ***x***.

Consequently, the nominal design ***x***^(0)^ is found by solving

4 $${\mathbf{x}}^{(0)} = \arg \mathop {\min }\limits_{{\mathbf{x}}} U\left( {{\mathbf{x}},f_{t} } \right)$$

where the objective function

5 $$U\left( {{\mathbf{x}},f_{t} } \right) = L_{0} \left( {\mathbf{x}} \right) + \beta \left( {f_{t} - f_{0} \left( {\mathbf{x}} \right)} \right)^{2}$$

Thus, the goal is to minimize the level *L*_0_ while ensuring that the resonant frequency equals to *f*_*t*_. Note that we have two objectives here, with the level being the primary one, and the resonant frequency allocation implicitly^41^ handled as an equality constraint. The second term in (5) is a penalty term that enforces satisfaction of the condition *f*_0_(***x***^(0)^) = *f*_*t*_^42^. Here, the problem (4), (5) is solved using the trust-region (TR) gradient-based algorithm^43^ with the filter sensitivities estimated using finite differentiation (FD)^44^. The TR algorithm produces a series ***x***^(*i*)^, *i* = 0, 1, …, of approximations to ***x***^(0)^

6 $${\mathbf{x}}^{(i + 1)} = \arg \mathop {\min }\limits_{{{\mathbf{x}};\;||{\mathbf{x}} - {\mathbf{x}}^{(i)} || \le d^{(i)} }} U_{L} ({\mathbf{x}})$$

The linearized objective *U*_*L*_ is defined similarly as in (5) but the with filter transmission *S*_21_ evaluated using the first-order Taylor model *S*_21*L*_^(*i*)^(***x***,*f*) = *S*_21_(***x***^(*i*)^,*f*) + ***G***_21_(***x***^(*i*)^,*f*)⋅(***x ***− ***x***^(*i*)^). As mentioned, the gradient ***G***_21_(***x***,*f*) of *S*_21_(***x***,*f*) at ***x*** and frequency *f* is evaluated using FD^44^. The search size *d*^(*i*)^ > 0 is adjusted using the TR rules^43^. The sub-problem (6) is solved using a Sequential Quadratic Approximation (SQP) algorithm^45^. More specifically its implementation available in Matlab Optimization Toolbox is employed^46^.

**Figure 4:**
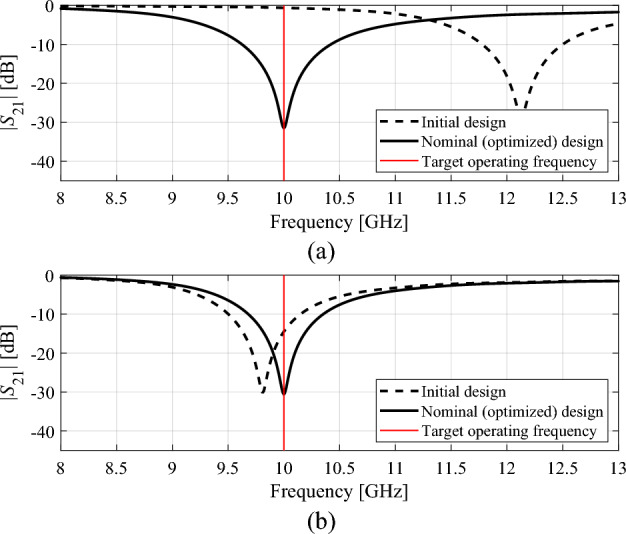
CSRR-based filters of "Resonator-based notch filters: case studies" section: initial and optimized nominal designs assuming target operating frequency *f*_*t*_ = 10 GHz: (**a**) circular filter, (**b**) square filter.

For the sake of illustration and subsequent considerations, the circular and square CSRR-based filters of "Resonator-based notch filters: case studies" section have been optimized assuming *f*_*t*_ = 10 GHz. The initial designs are ***x***_*init*_ = [0.5 0.8 1.1 1.4 0.3]^*T*^ and ***x***_*init*_ = [1.0 1.6 2.2 2.8 0.2]^*T*^ for the circular and square filters, respectively, whereas the nominal design found using (4), (3) are ***x***^(0)^ = [0.433 0.790 1.305 1.685 0.202]^*T*^ and ***x***^(0)^ = [0.956 1.563 2.152 2.791 0.221]^*T*^, respectively (dimensions in mm). The computational cost of the optimization process was 48 and 27 EM analyses of the respective circuit. Figure 4 shows the filter responses at the initial and nominal designs.

### Fabrication yield: definition and numerical estimation

Let ***x***^(0)^ be a nominal design of the CSRR-based filter as discussed in "Performance specifications: nominal design and design optimization" section. Recall, that ***x***^(0)^ has been obtained to allocate the notch at the target frequency *f*_0_ and to increase its depth *L*_0_ as much as possible. As indicated in "Filter prototyping: manufacturing tolerances" section, unavoidable manufacturing tolerances may lead to a severe misalignment between the simulation-predicted and experimentally-validated circuit response, especially in terms of the notch frequency shifts. In particular, the filter prototype may fail to fulfill the performance specifications imposed on its electrical characteristics. The purpose of statistical analysis and robust design is to quantify the effects of manufacturing inaccuracies, and to increase the likelihood that the specifications are satisfied under the assumed tolerance levels. Let *d****x*** stand for a vector of deviations of the circuit geometry parameters. Here, it is assumed that these deviations are a result of manufacturing imperfections, e.g., under/over etching in chemical processing pertinent to the PCB technology. In this work, it is assumed that *d****x*** follows joint Gaussian distribution with zero mean and variance *σ*, which is identical for all parameters. In general, the distribution may account for parameter correlations and described using an appropriate covariance matrix.

We also define an auxiliary function *H*(***x***), which is used to discriminate between designs that satisfy performance specifications (for which *H*(***x***) = 1) and those that do not (for which *H*(***x***) = 0). Going back to our notch filter, we would have

7 $$H({\mathbf{x}}) = \left\{ {\begin{array}{*{20}l} 1 \hfill & {\max \left\{ {f \in F:|S_{{21}} ({\mathbf{x}},f)|} \right\} \le L_{{\max }} } \hfill \\ 0 \hfill & {{\text{otherwise}}} \hfill \\ \end{array} } \right.$$

The primary statistical figure of merit is the fabrication yield *Y*, which is defined as follows. Let *p*(***x***,***x***^(0)^) be the probability density function describing deviations of the actual design ***x*** from the nominal one ***x***^(0)^. We have^47^

8 $$Y({\mathbf{x}}^{(0)} ) = \int\limits_{{X_{f} }} {p({\mathbf{x}},{\mathbf{x}}^{(0)} )d{\mathbf{x}}}$$

The feasible set *X*_*f*_ contains all designs that satisfy the performance specifications, i.e., ***x*** ∈ *X*_*f*_ if and only if *H*(***x***) = 1.

In practice, the feasible set is not known explicitly, therefore, the yield is estimated using numerical methods, in particular, Monte Carlo (MC) simulation. Let *d****x***^(*k*)^, *k* = 1, …, *N*_*r*_, be random designs randomly assigned using the density function *p*. The estimated yield is then obtained as

9 $$Y({\mathbf{x}}^{(0)} ) = N_{r}^{ - 1} \sum\nolimits_{k = 1}^{{N_{r} }} {H({\mathbf{x}}^{(0)} + d{\mathbf{x}}^{(k)} )}$$

where *H* is defined as in (7).

### Robust design and its challenges

A robust design task can be posed in various ways, e.g., as design centering, yield optimization, tolerance hypervolume maximization^26,29,33,48^. Notwithstanding, the overall objective is to increase the likelihood that the performance specifications imposed upon the circuit are satisfied under the assumed uncertainties, both aleatory (such as manufacturing inaccuracies)^21^ and epistemic (such as the lack of knowledge about operating conditions, etc.)^30^. Here, we are only interested in fabrication tolerances, and the robust design problem is formulated as yield maximization. Consequently, the optimum design is found by solving

10 $${\mathbf{x}}^{*} = \arg \mathop {\min }\limits_{{\mathbf{x}}} \{ - Y({\mathbf{x}})\}$$

As already mentioned in "Introduction" section, statistical design is a computationally expensive endeavour. On the one hand, numerical estimation of the statistical figures of merit such as yield (cf. "Performance specifications: nominal design and design optimization" section) is already expensive. For example, producing reliable results through MC simulation (cf. (5)) normally requires hundreds of EM analyses, whereas solving (5) entails a large number of yield estimations, which is prohibitive when carried out directly at the level of EM simulations. Consequently, the majority of practical robust design techniques involve fast surrogate models to expedite evaluation of the yield and its optimization. As indicated in "Introduction" section, behavioural models are typically used such as kriging, neural networks, or polynomial chaos expansion^20,22,31,34,49^. Notwithstanding, constructing reliable metamodels over multidimensional parameter space poses considerable challenges, and generally requires large amounts of training data, which is detrimental to the computational efficiency of the design process. In this work, in order to facilitate surrogate model construction of notch filters for the purpose of yield estimation, a response feature technology is incorporated^50^, as elaborated on in "Feature-based surrogate modeling of notch filters" section.

### Feature-based surrogate modeling of notch filters

Behavioral modeling of highly-nonlinear responses of notch filters is a challenging task, which requires considerable computational investments in terms of training data acquisition. In this work, we use a response feature technology to facilitate the modeling process, and reduce to cost of statistical analysis of the filters, and, subsequently, their yield optimization. Reformulating the modeling task in terms of appropriately defined characteristic points smoothens out the functional landscape to be handled, and makes a construction of a surrogated model considerably simpler^49,50^. The characteristic (or feature) points should account for design specifications. Given the condition (3) assumed in this paper, it is sufficient to consider the following vectors

11 $${\mathbf{p}}_{f} ({\mathbf{x}}) = [f_{L} ({\mathbf{x}})\;f_{H} ({\mathbf{x}})]^{T}$$

and

12 $${\mathbf{p}}_{L} ({\mathbf{x}}) = [L_{L} ({\mathbf{x}})\;L_{H} ({\mathbf{x}})]^{T}$$

where *f*_*L*_(***x***) and *f*_*H*_(***x***) are the frequencies corresponding to the filter bandwidth at the *L*_max_ level (cf. (3)) of the transmission characteristic |*S*_21_|. Correspondingly, *L*_*L*_(***x***) and *L*_*H*_(***x***) are both equal to *L*_max_. Thus, the vectors ***p***_*f*_ and ***p***_*L*_ stand for the frequency and level coordinates of the feature points. We will also use the notation

13 $${\mathbf{P}}({\mathbf{x}}) = [{\mathbf{p}}_{f} ({\mathbf{x}})^{T} \;{\mathbf{p}}_{L} ({\mathbf{x}})^{T} ]^{T}$$

The vector ***P***(***x***) is sufficient to determine whether the filter satisfies the performance condition (3) at design ***x***, which can be re-written in terms of the feature points as

14 $$f_{L} \left( x \right) \le f_{t} {-}B/{2}\quad {\text{AND}} \quad f_{H} \left( x \right) \ge f_{t} + B/{2}$$

**Figure 5:**
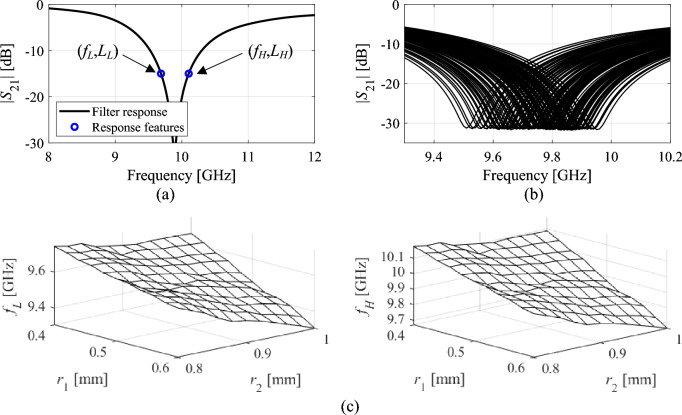
Response features of notch filters: (**a**) transmission responses and two feature points with the corresponding feature vectors ***p***_*f*_(***x***) = [*f*_*L*_(***x***) *f*_*H*_(***x***)]^*T*^ and ***p***_*L*_(***x***) = [*L*_*L*_(***x***) *L*_*H*_(***x***)]^*T*^, here, corresponding to *L*_max_ = − 15 dB; (**b**) family of filter responses corresponding to vectors ***x*** = [*r*_1_ *r*_2_ *r*_3_ *r*_4_ *s*]^*T*^ with 0.4 ≤ *r*_1_ ≤ 0.6, 0.8 ≤ *r*_2_ ≤ 1.0, *r*_3_ = 1.3, *r*_4_ = 1.7, *s* = 0.2 (all dimensions in mm); (**c**) frequency coordinates of the feature points corresponding to the same parameter space region. Observe that modeling of complete responses is incomparably more challenging than modeling of the feature point coordinates.

Figure 5a shows an exemplary response of a notch filter (here, the circular CSRR-based structure of "Resonator-based notch filters: case studies" section) along with the corresponding feature points. Further, Fig. 5b shows a family of filter responses evaluated over a selected region of the parameter space, here, ***x*** = [*r*_1_ *r*_2_ *r*_3_ *r*_4_ *s*]^*T*^ with 0.4 ≤ *r*_1_ ≤ 0.6, 0.8 ≤ *r*_2_ ≤ 1.0, *r*_3_ = 1.3, *r*_4_ = 1.7, *s* = 0.2 (all dimensions in mm), whereas Fig. 5c illustrates the frequency coordinates of the feature points of the same region. It can be observed that behavioral modeling of the feature point is considerably simpler than modeling of the complete filter responses, which is due to weakly-nonlinear dependence between ***p***_*f*_(***x***) and geometry parameter vector ***x***.

Given the aforementioned properties of response features, the feature-based surrogate considered in this work takes a simple form, which is a quadratic model without mixed terms, defined below. In Sect. "Surrogate-assisted yield estimation and optimization by feature-based models," we will be discussing an iterative yield optimization procedure, which produces a sequence of designs ***x***^(*i*)^, *i* = 0, 1, … (***x***^(0)^ being the nominal design of "Fabrication yield: definition and numerical estimation" section). The feature-based surrogates constructed in each iteration will be denoted as ***s***_*P*_^(*i*)^, and defined as

15 $${\mathbf{s}}_{P}^{(i)} ({\mathbf{x}},{{\varvec{\uplambda}}}) = \left[ \begin{gathered} \lambda_{L.0} + \sum\limits_{j = 1}^{n} {\lambda_{L.j} x_{j} } + \sum\limits_{j = 1}^{n} {\lambda_{L.n + j} x_{j}^{2} } \hfill \\ \lambda_{H.0} + \sum\limits_{j = 1}^{n} {\lambda_{H.j} x_{j} } + \sum\limits_{j = 1}^{n} {\lambda_{H.n + j} x_{j}^{2} } \hfill \\ \end{gathered} \right]$$

The coefficient vector ***λ*** = [***λ***_*L*_^*T*^ ***λ***_*H*_^*T*^]^*T*^, ***λ***_*L*_ = [*λ*_*L.*0_ … *λ*_*L.*2*n*_]^*T*^ and ***λ***_*H*_ = [*λ*_*H.*0_ … *λ*_*H.*2*n*_]^*T*^, is found by solving the regression problems

16 $${\mathbf{s}}_{P}^{(i)} ({\mathbf{x}}_{B}^{(k)} ,{{\varvec{\uplambda}}}) = {\mathbf{p}}_{f} ({\mathbf{x}}_{B}^{(k)} ),\quad k = 0,{1}, \ldots ,{2}n$$

where {***x***_*B*_^(*k*)^,***p***_*f*_(***x***_*B*_^(*k*)^)} are training pairs with ***x***_*B*_^(0)^ = [*x*_*B*.1_^(0)^ … *x *_*B*.*n*_^(0)^]^*T*^ = ***x***^(*i*)^ = [*x*_1_^(*i*)^ … *x*_*n*_^(*i*)^]^*T*^, ***x***_*B*_^(*k*)^ = [*x*_1_^(*i*)^ … *x*_*k*−1_^(*i*)^ *x*_*k*_^(*i*)^ + *d*_max_ *x*_*k*+1_^(*i*)^ … *x*_*n*_^(*i*)^]^*T*^ for *k* = 1, …, *n* (positive perturbations), and ***x***_*B*_^(*k*)^ = [*x*_1_^(*i*)^ … *x*_*k*−1−*n*_^(*i*)^ *x*_*k*−*n*_^(*i*)^ − *d*_max_ *x*_*k*+1−*n*_^(*i*)^ … *x*_*n*_^(*i*)^]^*T*^ f or *k* = *n* + 1, …, 2*n* (negative perturbations). Here, *d*_max_ = 3*σ* where *σ* was the joint variance of the Gaussian probability distribution assumed to govern the fabrication tolerances of the filter (cf. "Robust design and its challenges" section).

Because the problems (16) are linear regression tasks, the coefficients ***λ*** can be found analytically as

17 $${{\varvec{\uplambda}}}_{L} = {\mathbf{A}}^{ - 1} {\mathbf{F}}_{L}$$

and

18 $${{\varvec{\uplambda}}}_{H} = {\mathbf{A}}^{ - 1} {\mathbf{F}}_{H}$$

where

19 $${\mathbf{A}} = \left[ {\begin{array}{*{20}c} 1 & {x_{B.1}^{(0)} } & \cdots & {x_{B.n}^{(0)} } & {(x_{B.1}^{(0)} )^{2} } & \cdots & {(x_{B.n}^{(0)} )^{2} } \\ \vdots & \vdots & \ddots & \vdots & \vdots & \cdots & \vdots \\ 1 & {x_{B.1}^{(2n)} } & \cdots & {x_{B.n}^{(2n)} } & {(x_{B.1}^{(2n)} )^{2} } & \cdots & {(x_{B.n}^{(2n)} )^{2} } \\ \end{array} } \right]$$

20 $${\mathbf{F}}_{L} = \left[ \begin{gathered} f_{L} ({\mathbf{x}}_{B}^{(0)} ) \\ \vdots \\ f_{L} ({\mathbf{x}}_{B}^{(2n)} ) \\ \end{gathered} \right],\quad {\mathbf{F}}_{H} = \left[ \begin{gathered} f_{H} ({\mathbf{x}}_{B}^{(0)} ) \\ \vdots \\ f_{H} ({\mathbf{x}}_{B}^{(2n)} ) \\ \end{gathered} \right]$$

The mentioned weakly-nonlinear dependence between the feature point coordinates the geometry parameters of the notch filter makes the model (15) reliable despite being set up using only 2*n* + 1 training samples. Furthermore, its extrapolation capability is expected to be superior to that of conventional (e.g., kernel-based) surrogates such as radial-basis functions or kriging, let alone neural networks.

For illustration purposes, a feature-based surrogate (15) has been established for the circular CSRR-based filter of "Resonator-based notch filters: case studies" section at the nominal design ***x***^(0)^ = [0.344 0.790 1.305 1.685 0.202]^*T*^ discussed in "Fabrication yield: definition and numerical estimation" section. The assumed variance *σ* = 0.017 mm with the corresponding *d*_max_ = 0.05 mm, which are the figures corresponding to typical manufacturing tolerances for PCB technology (chemical etching). The assumed target level *L*_max_ = − 10 dB.

Table 1 shows the relative RMS error values for the feature-based surrogate ***s***_*P*_^(0)^(***x***) and the kriging interpolation models^51^, set up in the interval *X*^(0)^ = [***x***^(0)^ − *d*_max_, ***x***^(0)^ + *d*_max_] using 11, 20, 50 and 100 training data samples allocated using Latin Hypercube Sampling^52^. Here, kriging has been employed as a benchmark method as one of the most popular data-driven modeling approaches. The modeling error has been estimated using 50 independent testing points allocated in the interval *X*^(0)^ (to test generalization error of the surrogates), and then, in the interval [***x***^(0)^ − 4*d*_max_/3, ***x***^(0)^ + 4*d*_max_/3] \ *X*^(0)^ to test the extrapolation capability of the models within the region of the ‘thickness’ *d*_max_/3 out of *X*^(0)^. The latter will correspond to the local yield optimization domain discussed in "Feature-based surrogate modeling of notch filters" section. In the case of kriging, the surrogate has been established for the complete transmission characteristics (separately for the real and imaginary parts thereof); however, for fair comparison, the error has been evaluated for the feature point coordinates extracted from the model-predicted filter characteristics. As it can be observed, the feature-based surrogate model exhibits excellent generalization corresponding to only 0.17% of relative RMS error, which is significantly better than kriging model set up using the same number of training samples (eleven). As a matter of fact, conventional surrogate needs 100 training samples (cf. last row of Table 1) to match the predictive power of the feature-based model. In terms of extrapolation capability, which is important from the perspective of using the metamodel for yield optimization, the feature-based model exhibits by far the best performance of 1.5% of relative RMS error, which cannot be matched by conventional models even when using 100 training data samples.

**Table 1:** Predictive power of the feature-based and conventional surrogate models.

Modeling technique	Number of training samples	Generalization error^#^ (%)	Extrapolation error^$^ (%)
Feature-based surrogate	11	0.17	1.5
Kriging interpolation	11	2.44	4.8
	20	0.40	3.0
	50	0.20	2.4
	100	0.16	2.0

^#^ Testing samples allocated in the same domain *X*^(0)^ = [***x***^(0)^ − *d*_max_, ***x***^(0)^ + *d*_max_] as used to establish the model.

^$^ Testing samples allocated in the domain [***x***^(0)^ − 2*d*_max_, ***x***^(0)^ + 2*d*_max_] \ *X*^(0)^, which is external to the domain of model establishment.

**Figure 6:**
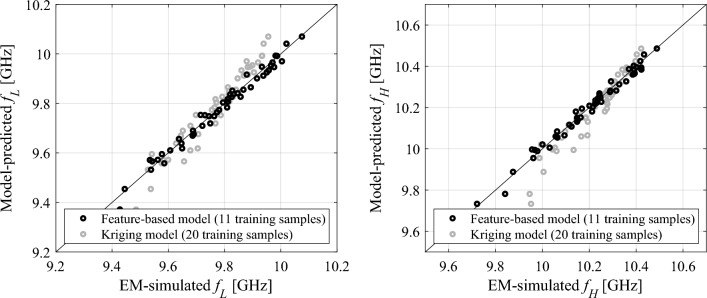
Scatter plots for feature-based surrogate model (15) constructed using 11 training samples for the circular CSRR-based notch filter at the nominal design ***x***^(0)^, assuming − 10 dB target level. Solid line represents an identify function, whereas circles represent 50 testing points. For comparison, gray circles show the scatter plot for kriging interpolation surrogates constructed using 20 training samples.

Figure 6 shows the scatter plots of the feature-based model for both frequencies *f*_*L*_ and *f*_*H*_. Excellent predictive power of the model can be observed.

### Surrogate-assisted yield estimation and optimization by feature-based models

The filter yield is optimized using the feature-based surrogate model (15), starting from the nominal design ***x***^(0)^. The optimization process is iterative, and the new iteration point is obtained by solving

21 $${\mathbf{x}}^{(i + 1)} = \arg \mathop {\min }\limits_{{{\mathbf{x}} \in X^{(i)} }} \{ - Y_{S}^{(i)} ({\mathbf{x}})\}$$

where the symbol *Y*_*S*_^(*i*)^ refers to the circuit yield estimated using the surrogate model ***s***_*P*_^(*i*)^(***x***) using the Monte Carlo process with 10,000 random samples generated using joint Gaussian distribution with the variance *σ*. The maximum deviation is limited to *d*_max_ = 3*σ*. The search domain is set to be an interval *X*^(*i*)^ = [***x***^(*i*)^ − *d*_max_/3, ***x***^(*i*)^ + *d*_max_/3]. It should be noted that evaluation of the yield *Y*_*S*_^(*i*)^(***x***) for any ***x*** ∈ *X*^(*i*)^ different than ***x***^(*i*)^ partially requires extrapolation. This is because the surrogate model domain is the interval [***x***^(*i*)^ − *d*_max_, ***x***^(*i*)^ + *d*_max_], whereas the yield estimation is based on the samples generated within the interval [***x*** − *d*_max_, ***x*** + *d*_max_]. However, as demonstrated in "Robust design and its challenges" section, the extrapolation capability of the feature-based metamodel is excellent within the considered region (cf. Table 1).

**Figure 7:**
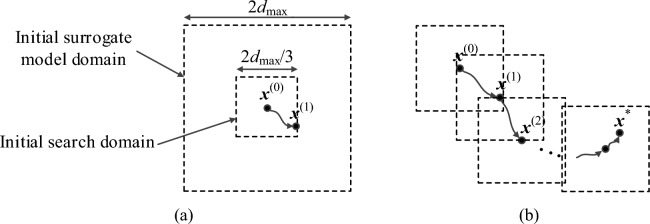
Yield optimization using feature-based surrogates: (**a**) nominal design, initial surrogate model domain (interval [***x***^(0)^ − *d*_max_, ***x***^(0)^ + *d*_max_]) and initial search domain (interval [***x***^(0)^ − *d*_max_/3, ***x***^(0)^ + *d*_max_/3]); (**b**) yield optimization with consecutive search domains marked as dashed-line intervals. A new feature-based surrogate model is constructed at each point ***x***^(*k*)^ as described in "Robust design and its challenges" section.

**Figure 8:**
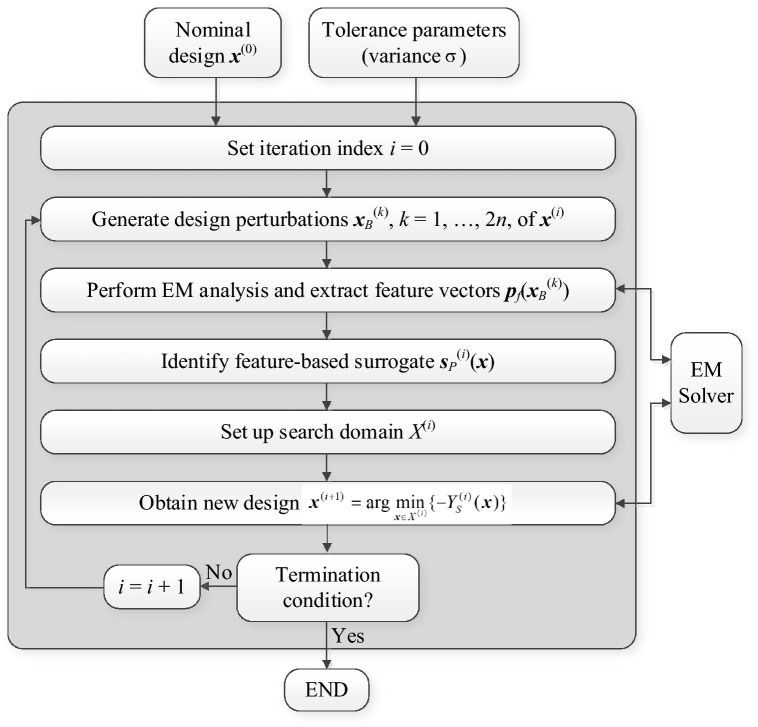
Flow diagram of the yield optimization process using feature-based surrogates.

The search process is terminated upon convergence in argument, i.e., when ||***x***^(*i*+1) ^− ***x***^(*i*)^||< *ε*, where *ε* = 10^−2^ for the experiments of "Demonstration examples" section. Figure 7 shows a graphical illustration of the initial surrogate model domain and the search region, as well as the progression of the search domains leading to the final design x^*^ = ***x***^(*i*+1)^, with *i* being the index of the iteration at which the optimization process was terminated. The flow diagram of the surrogate-assisted yield optimization process can be found in Fig. 8.

## Demonstration examples

This section discusses several demonstration examples that illustrate the operation and performance of the robust design procedure introduced in "Surrogate-assisted statistical design of notch filters using response features" section. We consider the circular and square CSRR-based notch filters introduced in "Resonator-based notch filters: case studies" section. In both cases, the nominal design is obtained for the target operating frequency of 10 GHz as elaborated on in "Performance specifications: nominal design and design optimization" section. The assumed variance for geometry parameter tolerances is *σ* = 0.017 mm, which corresponds to the maximum deviation of about 0.05 mm, consistent with PCB technology (chemical etching). The yield estimation and optimization results obtained using the feature-based surrogates are validated using EM-driven Monte Carlo analysis.

### Example I: circular CSRR-based notch filter

Our first demonstration example is the circular CSRR-based notch filter of "Resonator-based notch filters: case studies" section. The following two scenarios were considered:

- Target bandwidth *B* = 200 MHz with the target level *L*_max_ = − 15 dB;
- Target bandwidth *B* = 500 MHz with the target level *L*_max_ = − 10 dB.

In both cases, the center frequency is *f*_0_ = 10 GHz, and the nominal design ***x***^(0)^ = [0.433 0.790 1.305 1.685 0.202]^*T*^ mm is found as described in "Performance specifications: nominal design and design optimization" section. The yield-optimized designs are x^*.I^ = [0.499 0.709 1.253 1.757 0.256]^*T*^ and x^*.II^ = [0.493 0.694 1.230 1.772 0.242]^*T*^, for Case I and II, respectively. Table 2 shows the initial and final values of the fabrication yield predicted by the surrogate model, as well as validated using EM-based Monte Carlo analysis. Excellent agreement between surrogate-predicted and EM-simulated data can be observed. In both cases, the yield has been improved by about ten percent. One of the most important advantages of the presented approach is remarkable computational efficiency. The cost of the entire optimization process is lower than one hundred EM analyses. Table 2 also shows comparison with yield optimization using sequential approximate optimization and kriging interpolation as the surrogate modeling method. The kriging surrogate has been established in each iteration using 100 training samples, which ensures the accuracy comparable to the feature-based model (cf. Table 1), but with still noticeably worse extrapolation capability. Complete frequency responses of the filter are being modelled in this case. As it can be observed, the yield optimization results are generally similar in terms of the final yield values; however, the agreement between surrogate- and EM-simulation-predicted yield is not as good as for the feature-based model. Furthermore, the computational cost of the optimization process is significantly higher, by about an order of magnitude, due to a considerably larger size of the training dataset required to establish the model.

**Table 2:** Yield optimization and benchmarking results for circular CSRR-based notch filter.

Case	Surrogate modeling method	Design specifications		Initial yield		Optimized yield		Yield optimization cost^#^
		*B* [MHz]	*L*_max_ [dB]	Surrogate-predicted (%)	EM-driven MC simulation^$^ (%)	Surrogate-predicted (%)	EM-driven MC simulation^$^ (%)	
I	Feature-based (this work)	200	− 15	78	78	88	88	99
	Kriging interpolation			75	73	87	84	800
II	Feature-based (this work)	500	− 10	87	87	98	98	88
	Kriging interpolation			91	86	100	95	900

^$^ Monte Carlo simulation carried out using 500 random samples.

^#^ Cost expressed in terms of the number of EM simulations of the filter.

**Figure 9:**
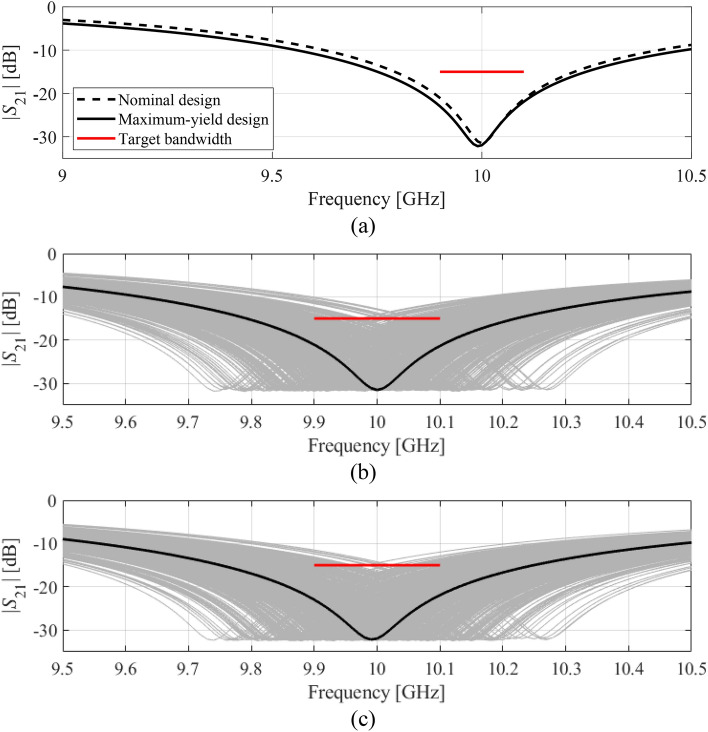
Yield optimization of circular CSRR-based notch filter, Case I: (**a**) transmission response at the nominal and optimized designs, (**b**) visualization of the EM-driven MC analysis at the nominal design, (**c**) visualization of the EM-driven MC analysis at the optimized design. The black lines represent the filter response at the nominal and the optimized designs, respectively, whereas the horizontal lines mark the target operating bandwidth.

**Figure 10:**
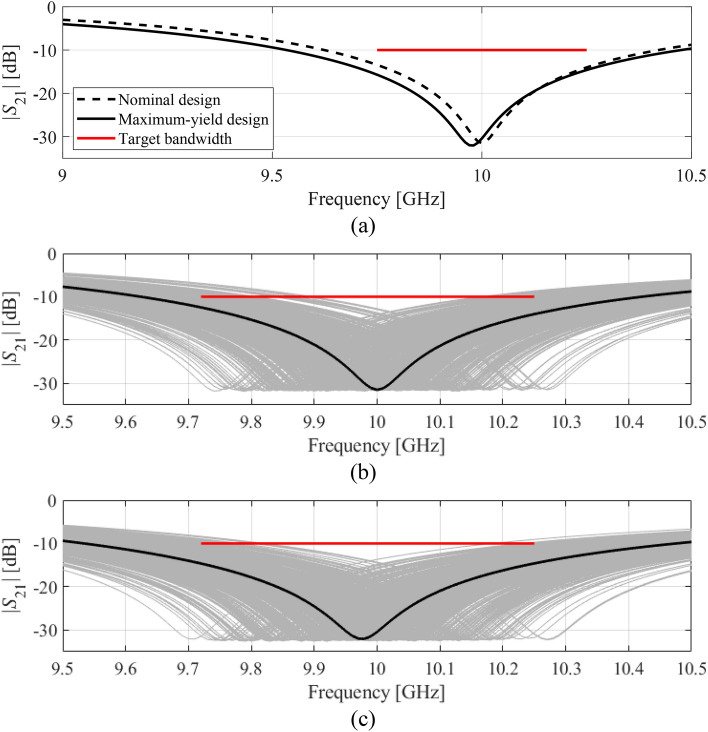
Yield optimization of circular CSRR-based notch filter, Case II: (**a**) transmission response at the nominal and optimized designs, (**b**) visualization of the EM-driven MC analysis at the nominal design, (**c**) visualization of the EM-driven MC analysis at the optimized design. The black lines represent the filter response at the nominal and the optimized designs, respectively, whereas the horizontal lines mark the target operating bandwidth.

Figures 9 and 10 show the filter transmission responses at the nominal and yield-optimized designs along with the visualization of EM-based Monte Carlo simulation for both considered cases. As it can be seen, the number of circuit responses violating design specifications is considerably smaller for the optimized designs as compared to the nominal ones, which is indicative of (visually) improved fabrication yield.

### Example II: square CSRR-based notch filter

**Figure 11:**
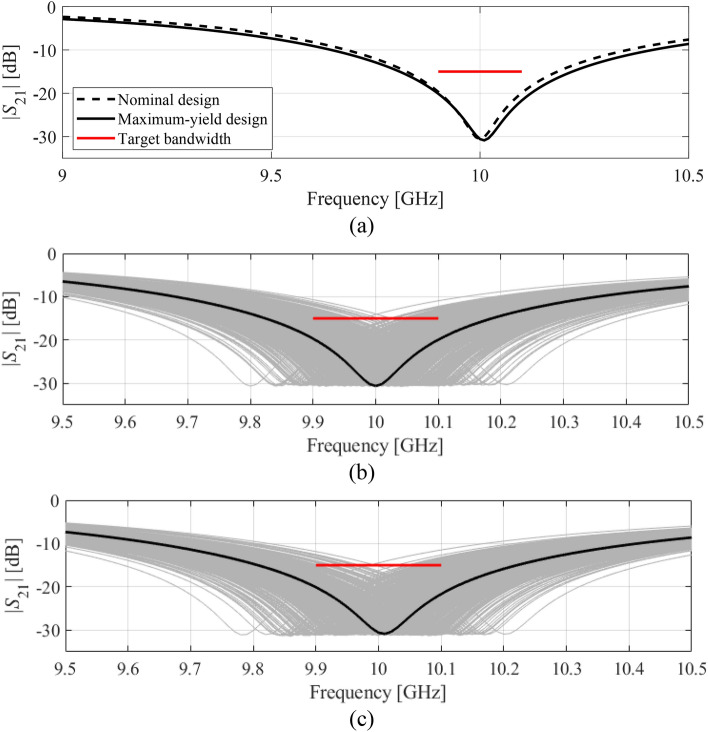
Yield optimization of square CSRR-based notch filter, Case I: (**a**) transmission response at the nominal and optimized designs, (**b**) visualization of the EM-driven MC analysis at the nominal design, (**c**) visualization of the EM-driven MC analysis at the optimized design. The black lines represent the filter response at the nominal and the optimized designs, respectively, whereas the horizontal lines mark the target operating bandwidth.

**Figure 12:**
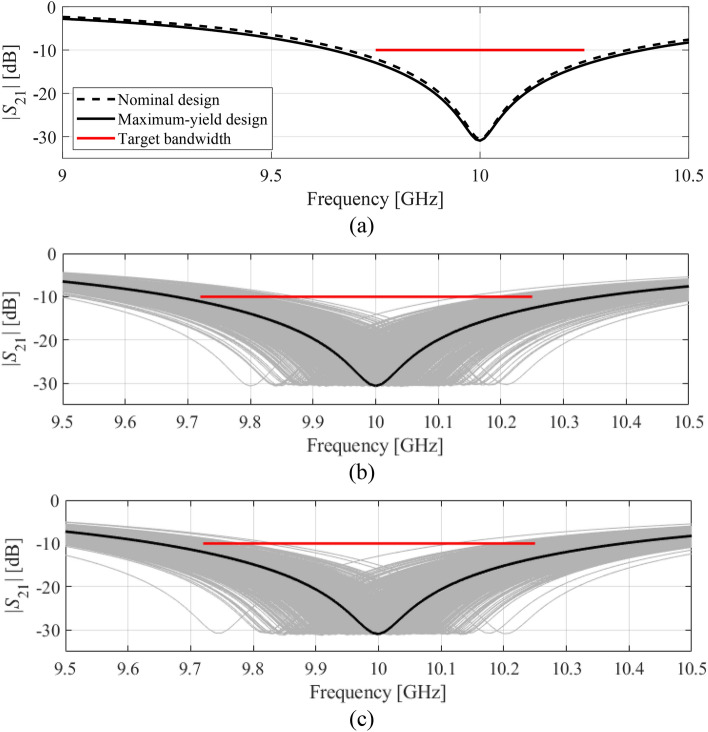
Yield optimization of square CSRR-based notch filter, Case II: (**a**) transmission response at the nominal and optimized designs, (**b**) visualization of the EM-driven MC analysis at the nominal design, (**c**) visualization of the EM-driven MC analysis at the optimized design. The black lines represent the filter response at the nominal and the optimized designs, respectively, whereas the horizontal lines mark the target operating bandwidth.

The second demonstration example is the square CSRR-based notch filter, also introduced in "Resonator-based notch filters: case studies" section. We consider the same scenarios as in "Example I: circular CSRR-based notch filter" section, i.e.: (1) target bandwidth *B* = 200 MHz with the target level *L*_max_ = − 15 dB; (2) target bandwidth *B* = 500 MHz with the target level *L*_max_ = − 10 dB. The center frequency is as before *f*_0_ = 10 GHz. The nominal design ***x***^(0)^ = [0.956 1.563 2.152 2.791 0.221]^*T*^ mm has been obtained as discussed in "Performance specifications: nominal design and design optimization" section. The yield-optimized designs are x^*.I^ = [9.997 1.429 2.120 2.879 0.204]^*T*^ and x^*.II^ = [0.945 1.479 2.145 2.863 0.214]^*T*^, for Case I and II, respectively. The initial and final values of the fabrication yield predicted by the surrogate model and corroborated through EM-based Monte Carlo analysis can be found in Table 3. Again, the agreement between the feature-model-predicted and EM-simulated data is excellent. The yield improvement is twelve percent for Case I, and fifteen percent for Case II. Similarly as for the circular filter, the optimization cost is very low and corresponds to only about a hundred of EM analyses on the average. The filter transmission responses at the nominal and yield-optimized designs as well as visualization of EM-based Monte Carlo simulation can be found in Figs. 11 and 12 for both considered cases. Similarly as for the previous example, the proposed method has been benchmarked against kriging interpolation as the surrogate modeling technique (cf. Table 3). Here, the kriging surrogate has been established in each iteration using 100 training samples to ensure fair comparison, i.e., similar predictive power of the kriging surrogate versus feature-based one. The results are consisted with those obtained for the first example: the yield optimization results are similar but the agreement between surrogate- and EM-simulation-predicted yield is noticeably degraded as compared with the feature-based model. Also, kriging-based optimization incurs considerably (almost ten-fold) higher computational expenses.

**Table 3:** Yield optimization and benchmarking results for square CSRR-based notch filter.

Case	Surrogate modeling method	Design specifications		Initial yield		Optimized yield		Yield optimization cost^#^
		*B* [MHz]	*L*_max_ [dB]	Surrogate-predicted (%)	EM-driven MC simulation^$^ (%)	Surrogate-predicted (%)	EM-driven MC simulation^$^ (%)	
I	Feature-based (this work)	200	− 15	77	78	89	89	110
	Kriging interpolation			80	77	91	87	800
II	Feature-based (this work)	500	− 10	78	80	93	94	77
	Kriging interpolation			75	78	97	92	800

^$^ Monte Carlo simulation carried out using 500 random samples.

^#^ Cost expressed in terms of the number of EM simulations of the filter.

### Discussion

The results presented in Table 1 through 3 allow us to make several observations concerning the performance of the proposed feature-based yield estimation and optimization procedure. These have been summarized below:

- Appropriate definition and utilization of characteristic points (here, the frequencies corresponding to the assumed target level of the transmission response) enables a considerable simplification and regularization of the yield estimation task, which carries over to yield optimization;
- Feature-based model exhibits remarkable predictive power while being established using a handful of training data samples (here, only 2*n* + 1 = 11). Analytically, the model is a low-order polynomial operating at the level of the feature points, which ensures excellent generalization but also extrapolation. The latter is instrumental for the reliability of the optimization process.
- When compared to conventional behavioral modeling, here, kriging interpolation, feature-based model can be set up using ten-fold smaller training datasets. This is essential for the efficiency of the optimization process, which requires rendition of multiple models.
- It can be noted that maximum-yield designs are not optimum in the nominal sense, as the resonant frequency of the filter is generally misaligned with respect to its target value (cf. Figures 9a, 10a, 11a, and 12a). Instead, the optimization process relocates the design so that the filter transmission response is adjusted in a more advantageous position (from the point of view of its deviations due to tolerances) at the target level *L*_max_. When changing *L*_max_ (Case I vs. Case II), the optimum design is adjusted accordingly.
- Yield estimation accuracy is excellent as corroborated using EM-driven Monte Carlo analysis based on 500 random samples. The differences are within one percent, which is actually less than the yield estimation variance by the MC process.
- The overall computational cost of yield optimization process is around one hundred EM analyses, which is an exceptionally low number in the context of robust design. As a matter of fact, this cost is comparable to that of conventional gradient-based optimization, and very much practical from the engineering perspective.
- Improvement of the fabrication yield, here, by ten to fifteen percent, depending on the considered case, greatly improves the likelihood that the assumed performance specifications will be satisfied given the underlying manufacturing tolerances.

Overall, the performance of the presented feature-based statistical design procedure is highly competitive. The algorithm itself is straightforward to implement and incorporated into conventional optimization routines.

## Conclusion

In this work, we introduced a simple yet powerful and computationally efficient procedure for statistical design of microstrip resonator-based notch filters. Our methodology capitalizes on the response feature approach, where the evaluation of the filter performance is carried out at the level of appropriately defined characteristics points of the system outputs. An accurate and analytically straightforward behavioral model is then constructed at the same level and utilized for rapid estimation of statistical figures of merit (here, the fabrication yield). A yield maximization procedure is developed by iteratively re-building the feature-based surrogate over local domain relocated along the optimization path. The operation and performance of the proposed approach has been demonstrated using two resonator-based notch filters, as well as a comparison of the modeling accuracy to the conventional methods working with complete filter responses. The obtained results corroborate a remarkably good predictive power of the feature-based metamodels, including their extrapolation capability. The models are constructed using a very small number of training data samples, which is linearly-dependent on the parameter space dimensionality. On the other hand, the computational cost of the yield optimization process corresponds to only about one hundred of EM simulations of the respective filter structure. At the same time, the yield estimation reliability is excellent, as validated through EM-driven Monte Carlo analysis.

Owing to its accuracy and computational efficiency, the statistical design procedure presented in this article can be considered a practical methodology for improving robustness of microstrip notch filter designs. It is straightforward to implement and handle, and it can be combined with various local search routines as optimization engines for solving local yield improvement sub-problems. The future work will be focused on expanding the applicability scope of the method, in particular, to include other types of uncertainties, e.g., pertaining to material parameters of the circuit substrate, as well as assembly inaccuracies.

A potential limitation of the proposed technique is that it requires the existence of the feature points across the entire yield estimation and optimization domain. This may become a problem if parameter deviations are excessively large, and the circuit responses are severely distorted. On the other hand, in the context of statistical design, this potential difficulty would not be as much pronounced because parameter deviations are normally reasonably small. Their sources are manufacturing imperfections, and, depending on the fabrication process, the tolerance levels are within the range of small fractions of millimeters. For example, the numbers assumed in "Demonstration examples" section (maximum deviation of 0.05 mm) are typical for chemical etching. In the case of mechanical or laser-based milling, the tolerances are even smaller, at the level of 0.01 mm or less. Furthermore, maintaining high values of yield throughout the optimization process implies that the circuit response will be not be distorted, which reduces the likelihood of feature disappearance.

## Data Availability

The datasets used and/or analysed during the current study available from the corresponding author on reasonable request.
